# A Restricted Two-Stage Multi-Locus Multi-Allele Genome-Wide Association Study Reveals Genomic Loci and Candidate Genes Controlling Plant-Height-Related Traits in Soybean Under Normal and Shade Conditions

**DOI:** 10.3390/ijms27125598

**Published:** 2026-06-20

**Authors:** Xiaoling Wu, Zhulian Chen, Rui Peng, Xinchun Liu, Jiajia Yang, Jingyi Ma, Chengxi Zhou, Dezhi Cai, Yanlin Liao, Xiaoli Chang, Jiang Liu, Weiguo Liu, Taiwen Yong, Feng Yang, Wenyu Yang

**Affiliations:** 1 College of Agronomy, Sichuan Agricultural University, Chengdu 611130, China; wuxl2014@163.com (X.W.); 15887957101@163.com (Z.C.);; 2 Sichuan Engineering Research Center for Crop Strip Intercropping System, Chengdu 611130, China; 3 College of Life Science, Sichuan Agricultural University, Yaan 625014, China

**Keywords:** soybean, plant height, RTM-GWAS, relay intercropping

## Abstract

Soybean is an important global crop used for oil, food, and feed production. To increase yield and land-use efficiency, growers often plant soybean at a high density or use intercropping systems. Under these systems, soybeans frequently experience shade stress, which directly affects agronomic traits such as plant height. Although researchers have well documented the genetic basis of plant height under normal conditions, the loci responsible for height variation under shade stress remain largely unexplored. Therefore, we performed a restricted two-stage multi-locus multi-allele genome-wide association study (RTM-GWAS) using SNP linkage disequilibrium block (SNPLDB) markers to identify QTLs associated with soybean plant height under shade stress. We evaluated a natural population of 181 soybean accessions for plant height traits under both normal and shaded conditions across four environments for three years. Using the Soybean40K chip, we derived 11,463 SNPLDB markers and identified 42, 33, and 28 significant SNPLDBs associated with plant height, average internode length, and number of main-stem nodes, respectively. For each SNPLDB, we estimated haplotype (allele) effects and assembled QTL–allele matrices to summarize the population’s genetic composition. Four SNPLDB loci proved stable across multiple environments, exhibiting high −lg(*p*) values and explaining substantial phenotypic variation. Finally, we projected that 80 candidate genes resided within 180 kb of these stable loci, and we identified four strong candidate genes linked to plant height traits based on combined positional and functional evidence. These results clarify genetic factors that influence soybean height under shading and could aid development of high-yielding soybean varieties.

## 1. Introduction

Soybean (*Glycine max* L.) has a documented domestication history of more than 5000 years, originating from wild soybean in China’s Huang-Huai-Hai region. Because soybean offers high-quality plant protein and oil, the crop has been widely cultivated in China and worldwide. Soybean provides grain, oil, and feed and therefore holds a central place in China’s agricultural production. Plant architecture constitutes a key target in breeding programs that aim to achieve super-high yields. Optimal morphological structures have been shown to increase yield substantially, as reported in previous studies [1]. The importance of crop architecture became especially clear during the first Green Revolution, when the adoption of short-stalked, high-yield cultivars produced large gains in productivity. A landmark advance in the 1950s identified and applied the semi-dwarf gene sd1 in rice; this gene moderately reduces plant height, improved lodging resistance, and raised yield potential. Shorter varieties yielded 16.67% more than taller counterparts in that work [2]. Because plant height in pod-bearing crops influences lodging resistance and the number of pods per plant, studies have shown that soybean height directly affects yield; consequently, genetic research on soybean plant height has become a central focus.

Soybean plant height is a complex quantitative trait influenced by various quantitative trait loci (QTLs). Numerous QTLs linked to soybean plant height have been identified. Mansur et al. (1993) reported a plant height QTL on the J (Chr 16) linkage group in the hybrid progeny of PI 27890 and PI 290136 [3]. Orf et al. (1999) identified three, four, and two QTLs controlling plant height in the recombinant inbred line populations of Minsoy × Noir1, Archer × Minsoy, and Noir1 × Archer, respectively [4]. Specht et al. (2001) found nine QTLs related to plant height traits in the recombinant inbred line (RIL) derived from the cross of Minsoy and Noir 1 [5]. Chapman et al. (2003) [6] analyzed plant height using the F_2_ and F_4:6_ populations constructed with Essex and Williams as parents. They identified a major plant height QTL locus, Satt540, on the M (Chr 7) linkage group in the F_2_ population, and another major QTL locus, Sat239, on the L (Chr 19) linkage group in the F_4:6_ population. Wang et al. (2004) identified six plant height QTLs on the C2 (Chr 6), E (Chr 15), K (Chr 9), M (Chr 7), and O (Chr 10) linkage groups using SSR markers in the BC_2_F_4_ population derived from IA2008 and PI468916 [7]. Kabelka et al. (2004) reported 12 plant height QTLs in the F_5_ population of BSR101xLG82-8379, located on the F (Chr 13), A1 (Chr 5), G (Chr 18), K (Chr 9), N (Chr 3), C2 (Chr 6), O (Chr 10), D1a (Chr 1), D1b (Chr 2), and H (Chr 12) linkage groups [8]. Liu et al. (2011) detected four plant height QTLs in two recombinant inbred line populations of Jinpumkong 2 × SS2-2 (J × S) and Iksannamulkong × SS2-2 (I × S) [9]. Lee et al. (2015) identified six plant height QTLs on the C1 (Chr 4), C2 (Chr 6), B1 (Chr 11), H (Chr 12), and F (Chr 13) linkage groups using the SNP genetic linkage map of the Wyandot x Pl567301B recombinant inbred line population, explaining 7% to 18% of the phenotypic variation [10]. Fang et al. (2020) identified four plant height QTLs in the Dongnong L13 × Henong 60 recombinant inbred lines, located on the C1 (Chr 4), M (Chr 7), F (Chr 13), and L (Chr 19) linkage groups, accounting for 10.48% to 19.55% of the phenotypic variation [11]. Tian et al. (2022) identified nine quantitative trait loci (QTLs) related to plant height in the recombinant inbred lines derived from Zhonghuang 13 × Zhongpin 03-5373 [12]. They discovered consistently expressed QTLs on chromosomes 13 and 19. Wang et al. (2022) identified two soybean plant height QTLs on chromosomes 5 and 14 through a combination of association analysis and QTL mapping [13]. Within the intervals flanking these QTLs, two potential candidate genes were identified.

Genome-wide association studies (GWASs) use statistical methods and molecular markers in natural or inbred populations to locate genes or loci that are significantly associated with specific traits. This approach offers high throughput, efficiency, and reliable results, making it a key tool for dissecting the genetics of complex plant traits [14,15]. In recent years, researchers have widely applied GWASs to investigate agronomic traits, stress resistance, and quality characteristics, and they have reported important discoveries. For example, GWASs have been used to identify genes and loci affecting yield and quality in rice, soybean, and maize, revealing multiple loci associated with yield, protein content, and oleic acid content [16,17,18,19,20]. Nevertheless, most GWAS findings come from general linear models (GLMs) and mixed linear models (MLMs). Statisticians have classified these approaches, which scan the genome by testing one marker at a time, as single-locus models [21,22]. Because single-locus analyses typically apply strict Bonferroni correction, they often miss loci that are truly linked to target traits [23]. In addition, when researchers study complex crop traits, traditional single-locus GWAS usually detect only a few major QTLs, which hinders comprehensive dissection of genome-wide QTL alleles [21,22]. Nonetheless, understanding the composition of genome-wide QTL alleles in germplasm resources is essential for molecular breeding.

To address the limitations of conventional single-locus GWASs in pinpointing QTLs for crop traits, recent studies have argued that multi-locus GWAS methods provide a more robust means of identifying genome-wide QTLs. He et al., (2017) proposed an innovative approach called the restricted two-stage multi-locus genome-wide association study (RTM-GWAS) [22]. The method estimates heritability to restrict the overall phenotypic variance attributable to loci and thereby improves the detection of genome-wide QTLs. RTM-GWAS combines a two-stage workflow with a multi-locus model to enhance QTL discovery across the genome. Researchers have applied this approach successfully to uncover genome-wide QTLs for diverse agronomic traits, stress tolerance, and quality attributes in natural and multi-parental soybean populations [21,24,25,26,27,28,29].

Soybean is a highly light-demanding crop. To expand planting areas and increase yields, farmers often intercrop or relay-intercrop it with maize and sorghum in some regions of China. Vandenbussche et al. (2005) reported that these practices create high-density stands with reduced light intensity and altered light quality, which produce a shaded environment [30]. As a result, soybeans in the lower canopy exhibit abnormal growth and development. For example, plant height, petiole length, hypocotyl length, and leaf shape index increase, whereas total lateral-root length and specific leaf weight decrease [31,32,33]. Flowering and pod-setting are delayed, and stems become thinner, elongated, and more branched, which promotes lodging [34,35,36]. Consequently, soybean yield and quality decline. Despite these effects, few studies have explored the genetic mechanisms underlying changes in soybean plant-height traits under shading stress. Building on prior work, this study subjected 181 soybean germplasm accessions to shading experiments in two environments over two years to identify the loci that control plant-height-related traits under normal light and shade condition. The results provide a theoretical and practical foundation for molecular breeding of high-yield soybean cultivars adapted to shade condition.

## 2. Result

### 2.1. Phenotypic Variations in Plant-Heigh-Related Traits Among Accessions

**Figure 1:**
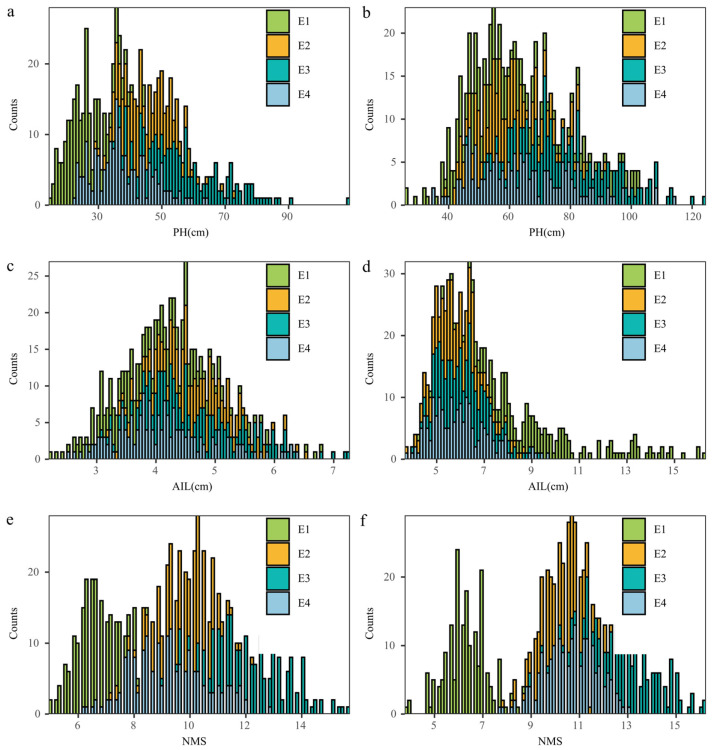
Phenotypic distributions of three plant-height-related traits among 181 soybean accessions in four planting environments (E1–E4). (**a**) PH under normal treatment, (**b**) PH under shade treatment, (**c**) AIL under normal treatment, (**d**) AIL under shade treatment, (**e**) NMS under normal treatment, (**f**) NMS under shade treatment. E1, E2, E3 and E4 denote Chongzhou in 2020, Renshou in 2021, Renshou in 2022 and Chongzhou in 2022, respectively.

Three height-related traits, plant height (PH), number of main stems (NMS), and average internode length (AIL), were assessed under normal light and shade conditions across four distinct environments (E1–E4) during the years 2020, 2021, and 2022. Under normal light (denoted as N), the PH, NMS, and AIL exhibited ranges from 14.82 to 108.83 cm, 5.00 to 15.67, and 2.22 to 7.25 cm, with respective averages of 41.67 cm, 9.47, and 4.33 cm across the four environments. Under shade conditions (denoted as S), the PH, NMS, and AIL ranged from 26.07 to 124.00 cm, 3.83 to 16.17, and 3.72 to 16.25 cm, with respective averages of 65.34 cm, 10.04, and 6.85 cm. The values of PH, NMS, and AIL were significantly higher under shade conditions compared to normal light. The coefficients of variation (CVs) for PH, NMS, and AIL under both normal and shade conditions demonstrated substantial phenotypic variability, with the CVs for PH being notably higher than those for NMS and AIL. The observed continuous and extensive phenotypic distributions indicate that these traits were characteristic of quantitative traits and suitable for genome-wide association studies (GWASs) (Table 1, Figure 1 and Table S2).

The broad-sense heritability (*h*^2^) of the three traits was calculated (Table 2). PH, NMS and AIL showed higher heritability under normal light than that under shade conditions. PH and AIL demonstrated high heritability (>60%) and substantial phenotypic variation under normal light. The analysis of variance (ANOVA) results indicated highly significant differences among genotypes, environments, treatments, and their interactions, suggesting that these traits were significantly influenced by environmental and treatment factors.

### 2.2. The LDB-Marker Construction and Population Structure

A total of 181 soybean accessions were sequenced using the soybean 40K liquid phase chip platform. The resulting sequence reads were aligned to the Williams82 reference genome version 1, leading to the identification of 139,499 SNP markers. Of these, 77,511 SNPs were retained using TASSEL 5.0, applying criteria of a deletion rate of ≤20% and a minor allele frequency (MAF) of ≥0.05. Chromosome 18 exhibited the highest number of SNPs (7093), whereas chromosome 12 exhibited the lowest (1814) (Table 3, Supplementary File S1). Additionally, the 77,511 SNPs were organized into 11,463 SNP linkage disequilibrium blocks (SNPLDBs), distributing all 20 chromosomes of the soybean genome (Table S3). These SNPLDBs comprised 3238 single SNP markers and 8225 multiple SNPs, accounting for 28.25% and 71.75% of the total, respectively. The 11,463 SNPLDBs contained 48,521 haplotypes/alleles, with each SNPLDB marker containing between 2 and 18 alleles; no SNPLDB markers with 1 or 17 alleles were observed (Table S4). The highest number of SNPLDBs was found on chromosome 18 (1040 SNPLDBs), while the lowest was on chromosome 12 (249 SNPLDBs).

**Figure 2:**
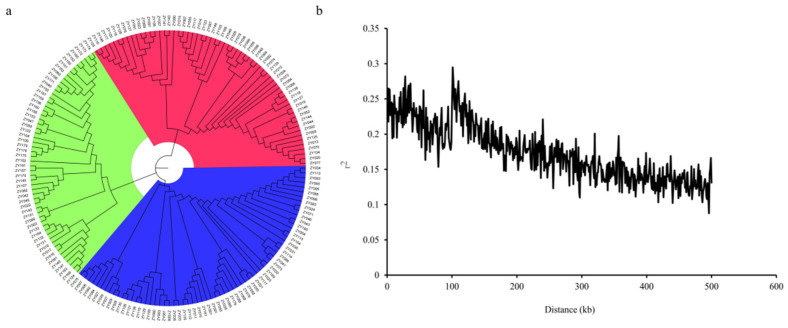
Population structure and LD decay based on SNPLDBs of 181 soybean accessions. (**a**) Phylogenetic tree of diversity panel. (**b**) Genome-wide mean decay of r^2^ in the population. Three different colors represent three distinct subpopulations.

The population structure of the 181 soybean accessions was estimated using the GSC matrix derived from whole-genome SNPLDB markers, and a phylogenetic tree was constructed based on genetic distances among these accessions. These 181 soybean accessions were classified into three distinct clusters (Figure 2a). Additionally, the linkage disequilibrium (LD) decay distance for the natural population was estimated to be approximately 180 kilobases (kb) for SNPLDBs, at which point the r^2^ value decreased to half of its maximum (Figure 2b).

### 2.3. GWASs for Plan-Height-Related Traits Under Normal Light and Shade Conditions

**Figure 3:**
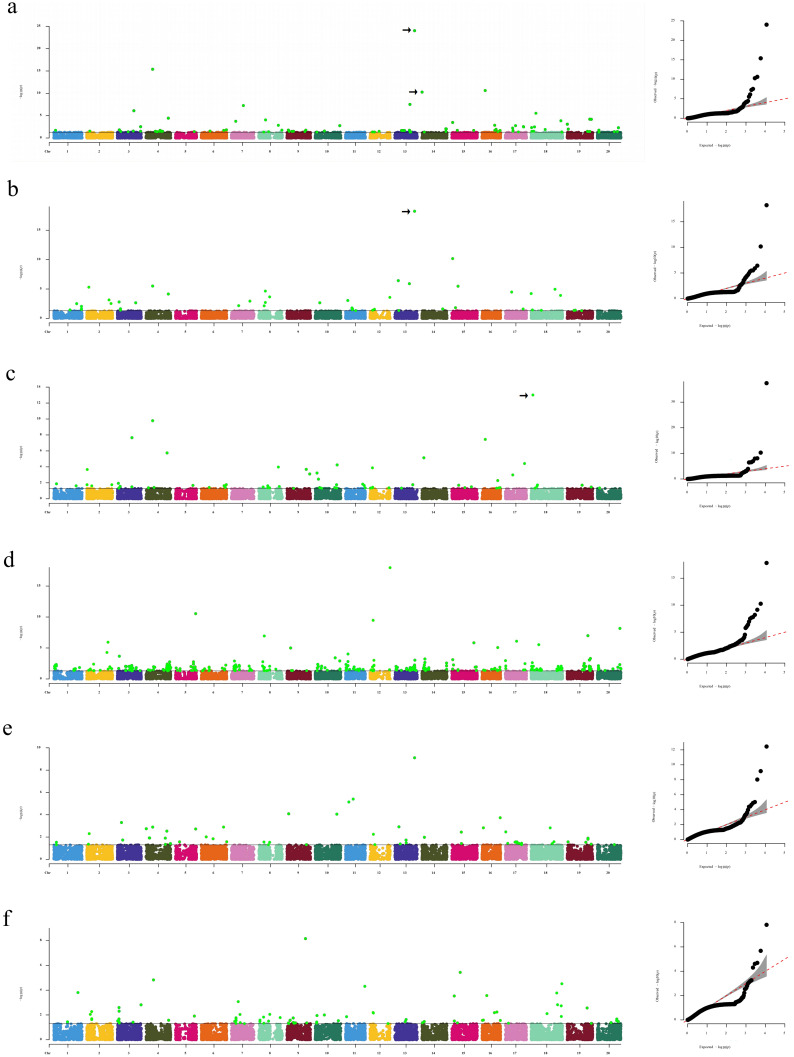
RTM-GWAS analyses of three plant-height-related traits among 181 soybean accessions under two kinds of shade treatment. (**a**–**f**) Manhattan (**left**) and quantile–quantile plots (**right**) of PH under normal treatment (**a**), PH under shade treatment (**b**), AIL under normal treatment (**c**), AIL under shade treatment (**d**), NMS under normal treatment (**e**), NMS under shade treatment (**f**), respectively. Each dot indicates one SNPLDB marker. The horizontal black lines represent the normal significance threshold of 0.05. The arrowed point represents the four stable SNPLDB loci. Each dot indicates one SNPLDB marker. The horizontal black lines represent the normal significance threshold of 0.05. The arrowed point represents the four stable SNPLDB loci.

The RTM-GWAS procedure, utilizing a multi-locus model, was employed to identify whole-genome QTL alleles linked to soybean height-related traits. A total of 42, 28, and 33 significant LDB loci were identified for PH, NMS, and AIL, respectively, using the multiple-environment RTM-GWAS approach (Table S5). Specifically, 52 and 51 LDBs were identified under normal light and shade conditions, respectively (*p* < 0.05). Of the 42 LDB loci associated with PH, seven SNPLDBs were found in at least one individual environment (Table 4). Notably, one SNPLDB locus (LDB_13_39157370_39256624) on chromosome 13 was consistently identified under normal light across the three environments (E1, E2, and E4), and under the shade condition across the three environments (E2, E3, and E4). This SNPLDB exhibited substantial −lg(*p*) values (24.03 and 18.23) and accounted for a notable percentage of phenotypic variation (1.70% and 2.14%) under normal light and shade conditions, respectively. Additionally, two SNPLDBs (LDB_14_228886 and LDB_13_39157370_39256624) demonstrated relatively high −lg(*p*) values (≥10.00) and are distinctly highlighted in Figure 3a,b. The remaining five SNPLDBs were detected in only one environment. Among the 28 significant SNPLDBs associated with NMS, 2 SNPLDBs (LDB_9_36844563 and LDB_18_61052742_61052) were identified under shade conditions in only one environment, and had a small −lg(*p*) value with a low PVE value. In the 33 SNPLDBs associated with AIL, 3 SNPLDB loci were also detected under normal light in one environment. Moreover, The SNPLDB LDB_18_3424413_3513390 was detected in only E4, with a −lg(*p*) value of 13.01 and a PVE value of 1.53%. The LDBs are labeled in Figure 3c.

### 2.4. Allele Effect of Significant SNPLDBs for the Plant-Height-Related Traits

**Figure 4:**
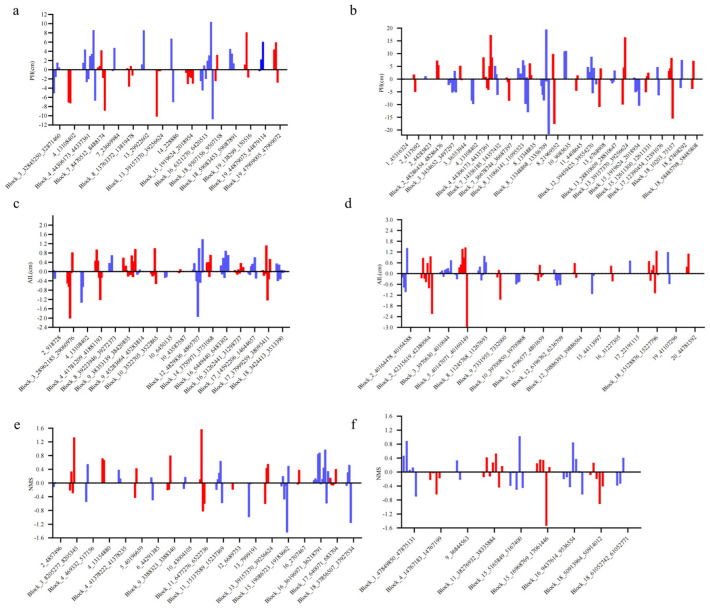
The distribution of allelic effects of the significant SNPLDB loci for three plant-height-related traits among 181 soybean accessions under two kinds of shade treatment. (**a**) PH under normal treatment, (**b**) PH under shade treatment, (**c**) AIL under normal treatment, (**d**) AIL under shade treatment, (**e**) NMS under normal treatment, (**f**) NMS under shade treatment. The different colored bars represent different SNPLDB loci.

The investigation examined the genetic influence of significant SNPLDB loci on plant-height-related traits under different light conditions. Allele effects were analyzed using RTM-GWAS v 1.4. Sixteen significant SNPLDB loci related to PH were identified under normal light, while 26 loci were found under shade conditions. In this study, it was observed that 25% of the loci were single SNPLDBs under normal light, whereas 50% were single SNPLDBs under shade conditions. The remaining loci were composed of multiple SNPLDBs. Through stepwise regression analysis, hereditary effects were identified across 221 alleles in 42 SNPLDB loci associated with plant height (PH) under both light conditions. Among these alleles, 111 exhibited positive effects, while 110 exhibited negative effects. The positive effects ranged from 0.01 to 23.06 cm, with an average of 5.30 cm, whereas the negative effects ranged from −33.34 to −0.03 cm, with an average of −5.35 cm (Figure 4a,b). Furthermore, 33 significant SNPLDB loci were associated with an average internode length (AIL), with 17 loci linked to normal light and 16 loci linked to shade conditions. Of these, 12 loci comprised multiple SNPLDBs, while 21 were single SNPLDBs. The positive effects of 113 alleles ranged from 0.01 to 2.96 cm, averaging 0.61 cm, while the negative effects of 133 alleles ranged from −2.96 to −0.01 cm, with a mean of −0.52 cm for single SNPLDBs (Figure 4c,d). In the context of NMS, 28 significant SNPLDB loci were identified, 19 multiple SNPLDBs and 9 single SNPLDBs under both light conditions. Single SNPLDBs accounted for 42.11% of the loci under normal light and 11.11% under shade conditions. The majority of SNPLDBs were multiple, representing 57.89% under normal and 89.89% under shade. A total of 78 positive alleles and 82 negative alleles were identified for NMS. Positive alleles ranged from 0.02 to 1.78, with a mean of 0.48, while negative alleles ranged from −0.01 to −2.23, with a mean of −0.46 (Figure 4e,f). Interestingly, haplotypes or alleles with extreme effects did not necessarily correspond with SNPLDB loci characterized by the highest −lg(*p*) values or the greatest percentages of explained phenotypic variation.

### 2.5. SNPLDB–Allele Matrices of Plant-Height-Related Traits in an Association Population

**Figure 5:**
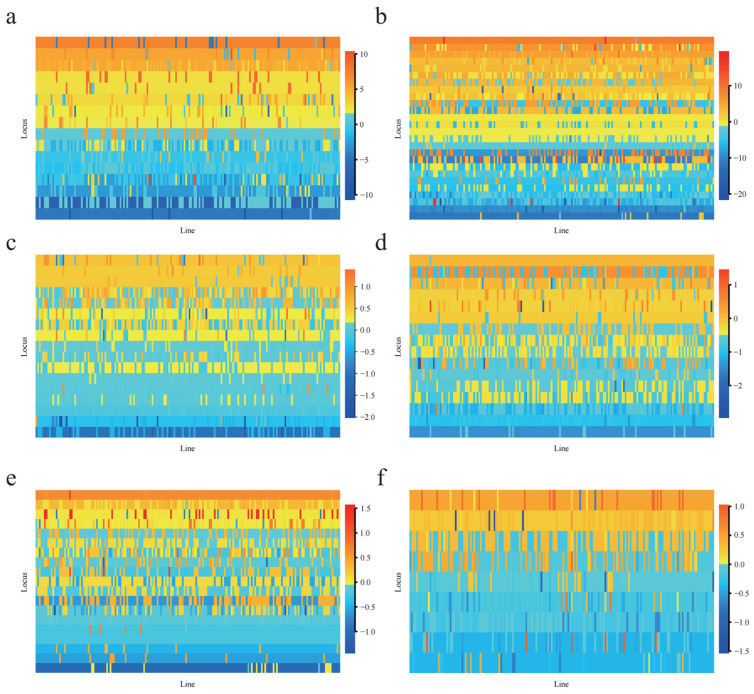
The QTL allele matrices for three plant-height-related traits among 181 soybean accessions under two kinds of shade treatment. (**a**) PH under normal treatment, (**b**) PH under shade treatment, (**c**) AIL under normal treatment, (**d**) AIL under shade treatment, (**e**) NMS under normal treatment, (**f**) NMS under shade treatment. The 181 soybean accessions are depicted on the horizontal axis, while the vertical axis displays the haplotypes or alleles of the significant SNPLDB loci. Red or yellow cells indicate positive alleles, and blue cells represent negative alleles, with color intensity reflecting the allele effect size. In the matrices, red or yellow cells signify positive alleles, blue cells denote negative alleles, and color intensity indicates the allele effect size.

The SNPLDB–allele matrices for three traits associated with plant height in 181 soybean accessions are illustrated in Figure 5a–f. Allele effects are represented by varying color intensities, with positive haplotypes/alleles depicted in red and yellow, and negative ones in blue. The depth of color indicates the magnitude of each haplotype or allele effect. For PH, 16 significant SNPLDBs were identified among the 181 accessions under normal light. These SNPLDBs were organized into a 16 × 181 matrix, providing a comprehensive view of the genetic composition related to PH, encompassing all significant SNPLDBs and their allele effects (Figure 5a). Similarly, 26 significant SNPLDBs associated with PH under shade conditions were arranged into 26 × 181 matrices, referred to as SNPLDB–allele matrices for PH under shade conditions (Figure 5b). An analogous approach was employed for SNPLDB loci associated with AIL, resulting in 33 × 181 matrices under both normal light and shade conditions, with 17 loci identified under normal light and 16 loci under shade conditions (Figure 5c,d). Furthermore, the effects of 19 and 9 identified SNPLDB loci related to NMS were outlined in 19 × 181 and 9 × 181 matrices under normal light and shade conditions, respectively (Figure 5e,f). Using the SNPLDB allele matrices, we identified both favorable and unfavorable alleles in each germplasm. These matrices also facilitated genetic diversity and richness at individual loci within the population. As a result, comprehensive allele frequency data derived from the SNPLDBs were available for further analysis.

### 2.6. Mining of FHs/FAs and Determination of the Stable SNPLDB Loci

**Figure 6:**
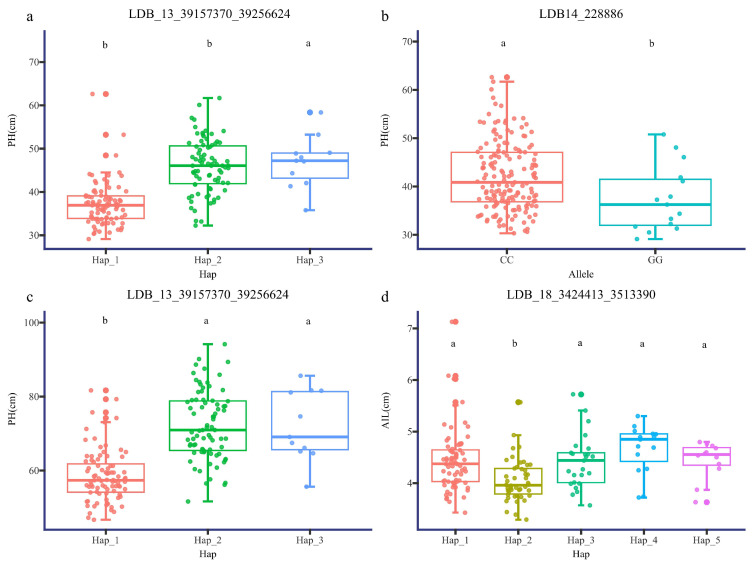
Phenotypic differences in the four major SNPLDB loci among different haplotypes or alleles. (**a**) LDB_13_39157370_39256624 for plant height (PH) under normal treatment, (**b**) LDB__14_228886 for plant height (PH) under normal treatment, (**c**) LDB_13_39157370_39256624 for plant height (PH) under shade treatment, (**d**) LDB_18_3424413_3513390 for average internode length (AIL) under normal treatment. The different lowercase letters indicate significant differences (*p* < 0.05) between different SNPLDB loci.

To further identify favorable haplotypes (FHs) or favorable alleles (FAs) of SNPLDBs associated with target traits, we concentrated on four key loci. These loci exhibited high −lg(*p*) values (−lg(*p*) ≥ 10) across multiple environments and were deemed crucial due to their significant impact on the traits under investigation. The haplotypes and alleles of these loci are illustrated in Figure 6a–d. Among the major SNPLDBs influencing PH under normal light, the locus LDB_13_39157370-39256624 revealed three distinct haplotypes: Hap_1 (CTACCTCTTCTCTTATGC), Hap_2 (CCCATCTCGTCTCCGTAC) and Hap_3 (TTACCTCTTCTCTTAGGT). Notably, the PH of 87 accessions with the positive haplotype Hap_1 was significantly lower than that of 90 accessions with the negative haplotypes Hap_2 and Hap_3 under normal light (Figure 6a, Table S6). Under shade conditions, the average PH of 87 accessions carrying Hap_1 was significantly lower than that of accessions with the other two haplotypes at SNPLDB locus LDB_13_39157370_39256624 (Figure 6c, Table S6). At the SNPLDB locus LDB_14_228886, two alleles (CC and GG) were identified. Accessions with the CC allele had a notably higher PH than those with the GG allele under normal light (Figure 6b, Table S6). Additionally, the SNP locus LDB_18_3424413_3513390 exhibited five haplotypes: Hap_1 (TCCCTAGCCA), Hap_2 (TCCCTAATCA), Hap_3 (TCCCTAGTCA), Hap_4 (GTTTCTGTGC), and Hap_5 (TCCCTAACCA). Accessions with Hap_2 (TCCCTAATCA) had significantly shorter average PHs than those with other haplotypes under normal light (Figure 6d, Table S6). Remarkably, the locus LDB_13_39157370_39256624 (Hap_1: CTACCTCTTCTCTTATGC) was a significant factor influencing PH under both normal and shade conditions. The loci LDB_13_39157370_39256624 for PH under normal and shade conditions, LDB_14_228886 for PH, and LDB_18_3424413_3513390 for AIL under normal light were selected due to their significant phenotypic differences between favorable and unfavorable allelic types. These findings underscore the importance of these SNPLDBs as key genetic loci affecting plant-height-related traits, specifically PH and AIL.

### 2.7. Distribution Frequency of FHs/FAs for the Four Stable SNPLDB Loci in Soybean Accessions

To validate the effects of FHs or FAs at the four stable SNPLDB loci on specific traits, we analyzed accessions with extreme phenotypic values, focusing on the top 30 and bottom 30 groups. We aimed to assess the cumulative occurrence of FHs/FAs for these traits.

**Figure 7:**
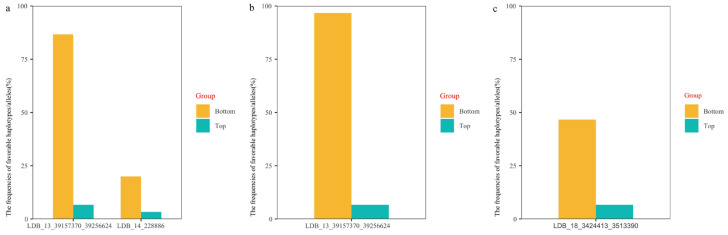
The frequencies of favorable haplotypes/alleles (FHs/FAs) for the four major SNPLDB loci. (**a**) The frequencies of FHs/FAs for 30 genotypes with the lowest PH and the 30 genotypes with the highest PH under normal treatment. (**b**) The frequencies of FHs/FAs for 30 genotypes with the lowest PH and the 30 genotypes with the highest PH under shade treatment. (**c**) The frequencies of FHs/FAs in 30 genotypes with the lowest AIL and the 30 genotypes with the highest AIL under normal treatment.

The results indicated that the frequency of FHs/FAs was notably higher in the bottom 30 accessions compared to the top 30 for two FHs/FAs (LDB_13_39157370_39256624-Hap1 and LDB__14_228886-GG), related to PH under normal light (Figure 7a). Similarly, the frequency of FHs was significantly elevated in the bottom 30 accessions compared to the top 30 for two pivotal SNPLDB loci, LDB_13_39157370_39256624-Hap1 and LDB_18_3424413_3513390-Hap2, associated with PH under shade conditions and for AIL under normal light, respectively (Figure 7b,c). This analysis of FH/FA frequencies at the major SNPLDB loci in soybean accessions highlights that FHs or FAs are more prevalent in shorter soybean accessions than in taller ones.

### 2.8. Identification of Potential Candidate Genes Associated with Plant-Height-Related Traits

**Figure 8:**
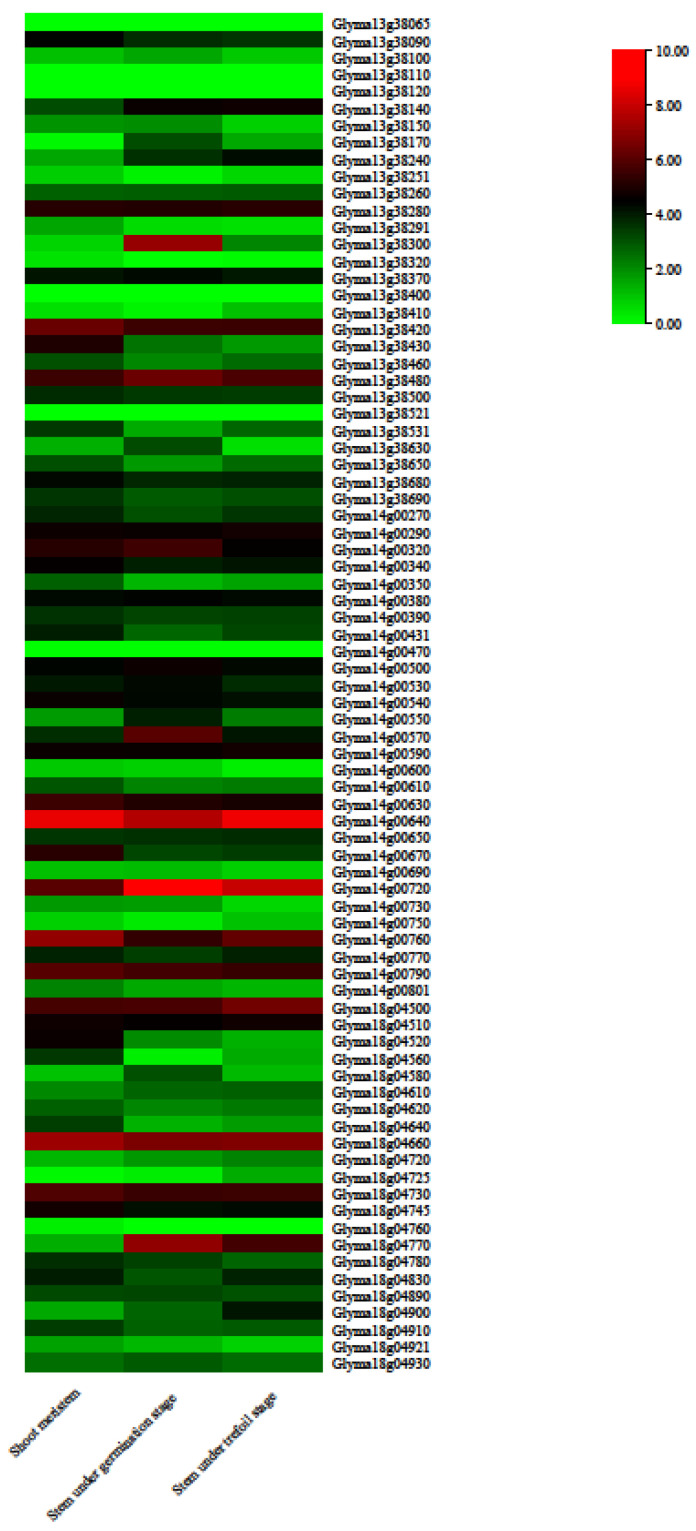
Heatmap of expression patterns of the 80 putative candidate genes among 3 soybean shoot-related tissues by the RNA-seq data from the SoyMD database (https://yanglab.hzau.edu.cn/SoyMD (accessed on 21 May 2024)). Red represents high expression, and blue represents low expression.

**Figure 9:**
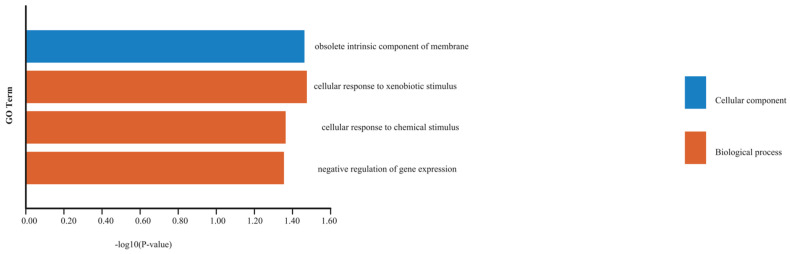
GO term classification for 23 genes in four major SNPLDB locus intervals.

To identify potential candidate genes associated with PH and AIL under normal light and shade conditions, we concentrated on specific stable SNPLDB loci: LDB_13_39157370_39256624, LDB_14_228886, and LDB_18_3424413_3513390. These loci were selected based on their relevance to plant height traits and their stability across different environments. A total of 80 genes were identified within a genomic region of ±180 kb surrounding the three SNPLDBs (Table S7). The distribution of these genes was as follows: 29 genes were located within the genomic interval of 38.97–39.43 Mb on chromosome 13, 29 genes within 0.07–0.43 Mb on chromosome 14, and 22 genes within 3.27–3.68 Mb on chromosome 18. Analysis of gene expression levels, using Fragments Per Kilobase of transcript per Million mapped reads (FPKM) values derived from RNA-Seq data, revealed that 74 out of the 80 genes were expressed in at least one of the three plant-height-related tissues (Figure 8). Subsequently, we conducted a Gene Ontology (GO) enrichment analysis using these 74 genes. Among them, 23 genes (31.08%) were annotated with respect to four specific terms in the agriGO database (https://systemsbiology.cau.edu.cn/agriGOv2/ (accessed on 20 May 2024)) (Figure 9), and one gene can be linked to multiple functional terms, underscoring the intricate nature of plant-height-related traits influenced by genes participating in diverse biological processes. By employing gene annotations, an in silico analysis, an RNA-seq analysis and a literature review, four specific genes, situated within three stable SNPLDB loci as potential candidates responsible for orchestrating plant height regulation, were identified in soybean. These genes, namely *Glyma14g00530* and *Glyma18g04580*, were positioned within a ±180 kb physical range of the stable SNPLDB loci LDB_14_228886 and LDB_18_3424413_3513390, respectively. *Glyma13g38500* and *Glyma13g38630* were located within LD decay intervals of the LDB_13_39157370_3925662 locus, which was mapped to chromosome 13. To validate their functional roles in governing plant height and internode length in soybean, further investigations such as knockout or overexpression experiments are imperative.

### 2.9. qRT-PCR Validation of Selected Candidate Genes

**Figure 10:**
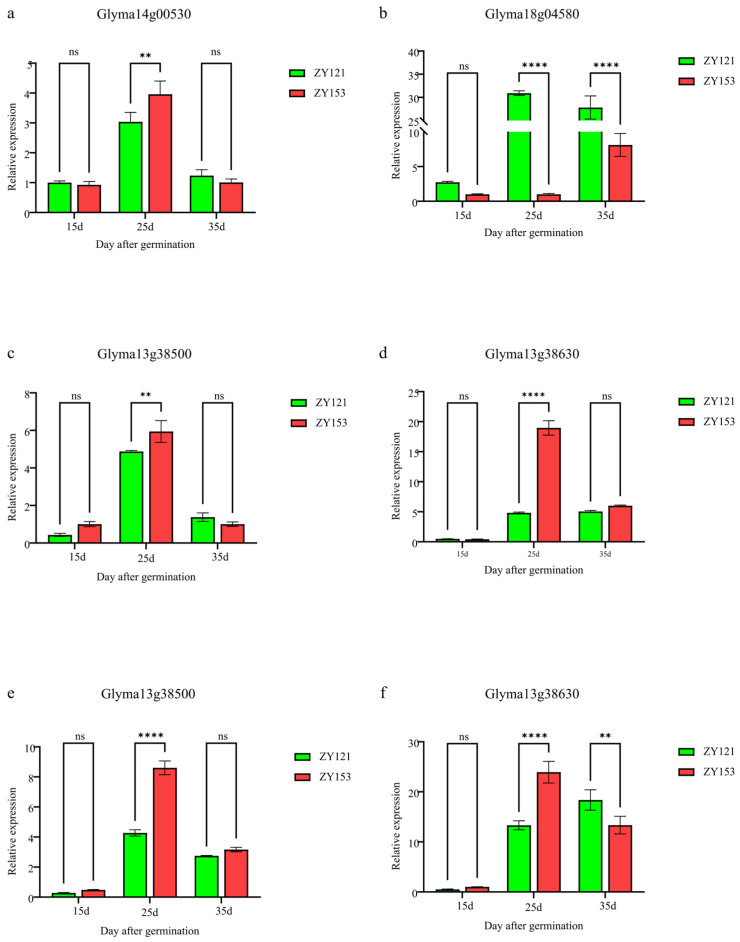
Expression patterns of candidate genes associated with plant-height-related traits under normal light and shade stress. The expression patterns of four candidate genes in two varieties at different times under normal light are illustrated: *Glyma14g00530* (**a**), *Glyma18g04580* (**b**), *Glyma13g38500* (**c**), and *Glyma13g38630* (**d**). The expression patterns of *Glyma13g38500* (**e**) and *Glyma13g38630* (**f**) genes in ZY153 (higher PH) and ZY121 (lower PH) at different times under shade stress. **, **** denote statistically significant differences at *p* < 0.01, *p* < 0.0001, respectively. ns denotes no statistically significant differences at *p* = 0.05 level.

To confirm the putative genes, we performed qRT-PCR analysis to evaluate the expression levels of four candidate genes (*Glyma13g38630*, *Glyma13g38500*, *Glyma14g00530*, and *Glyma18g04580*) in two soybean varieties: ZY121, which had a lower plant height (PH), and ZY153, which had a higher PH (Figure 10). Both varieties showed continuous growth in plant height (PH) and average internode length (AIL) from 15 to 35 days after germination under normal light conditions, as detailed in Supplementary Table S10. Notably, when subjected to shade stress, the PH characteristics of the two varieties remained consistent with those under normal light conditions (Table S9). Furthermore, our findings revealed a significant increase in PH and AIL for both varieties at 25 days after germination, irrespective of light conditions. This indicates that the 25-day mark is crucial for observing variations in PH and AIL of the experimental materials under varying light conditions (Table S9). The Relative Expression Level (REL) of *Glyma14g00530* consistently increased from 15 d to 25 d under normal light conditions, then decreased from 25 d to 30 d (Figure 10a, Table S10). The REL from 25 d was significantly higher in ZY153 than in ZY121, suggesting *Glyma14g00530* might act as a positive regulator in soybean plant height response. In contrast, Glyma18g04580 showed much lower expression in ZY153 at both 25 d and 35 d compared to ZY121 (Figure 10b, Table S10), indicating it might serve as a negative regulator in AIL. Additionally, two candidate genes, *Glyma13g38500* and *Glyma13g38630*, exhibited similar expression patterns under both normal light and shade stress (Figure 10c–f, Table S10). These genes had significantly higher expression levels in ZY153 at 25 d compared to those in ZY121. These findings suggest that the expression profiles of these genes are consistent with their proposed functions.

## 3. Discussion

Accurate phenotypic assessment plays a crucial role in the successful mapping of QTL and GWASs in crop research. In this investigation, three plant-height-related traits, PH, AIL and NMS, were meticulously analyzed across two distinct conditions in varying environments over a span of three years in soybean. The phenotypic data revealed a wide spectrum of variation among the traits studied. Soybeans often encounter shade stress in intercropping. Under shade conditions, soybean plants exhibit significant elongation in plant height, hypocotyl length, and first pod height, while concurrently experiencing reductions in branch number, pod number, node number, and grain weight per plant. These morphological changes collectively pose a substantial challenge to soybean productivity [32,33,36]. Precise phenotypic testing is indispensable for identifying superior gene pools essential for enhancing soybean shade tolerance in breeding programs.

The present study observed that soybean PH and AIL under shade conditions were significantly greater than those under normal light. The heritability (*h*^2^*)* of a trait, defined as the proportion of genetic variance relative to the total phenotypic variance, serves as a valuable metric for assessing the interplay between genetic and environmental factors, and directly influences the efficacy of association mapping analyses [37]. In this study, it was observed that the combined heritability estimates (*h*^2^) for PH and AIL were similar under normal and shade conditions. Conversely, the combined heritability for NMS was lower under normal light than that under shade.

Genome-wide association studies (GWASs), leveraging linkage disequilibrium and high-throughput sequencing technologies, proved to be an accurate method for QTLs in natural populations [38,39,40]. The initial standard for GWASs was the mixed linear model (MLM), a single-locus approach that effectively identifies several major loci associated with target traits, though it was also prone to generating false positives in some cases [41,42]. The RTM-GWAS based on a multiallelic model demonstrated superior power and efficiency in QTL discovery. Compared to the MLM-GWAS method, RTM-GWAS excelled in identifying a greater number of stable and genuine QTLs in both natural and bi-parental populations [22,43,44,45]. Given the RTM-GWAS procedure’s advantages in determining the complete spectrum of QTL alleles, we employed this method to uncover QTL alleles associated with soybean plant-height-related traits under shaded and normal conditions. In this study, SNPLDB loci were considered significant if they were consistently detected across multiple environments or detected in at least one environment with both high statistical significance (−lg(*p*) ≥ 10.00) and a substantial proportion of phenotypic variance explained (PVE ≥ 1.00%). These loci were considered major stable SNPLDB loci for plant-height-related traits. Through the multiple-environment RTM-GWAS procedure, a total of two significant SNPLDB loci associated with plant height were identified under both normal and shade treatments. In contrast, our previous research using the MLM-GWAS method detected only 11 significant SNP loci for plant height. Similarly, the RTM-GWAS procedure identified one stable AIL, compared to those not detected by the MLM-GWAS. These findings demonstrated that the RTM-GWAS method was more effective than the MLM-GWAS in identifying a greater number of QTLs for the targeted traits. In addition, The RTM-GWAS procedure provided a detailed QTL–allele matrix that captured essential genetic information, including significant SNPLDB loci, allele effects, population allele distributions, and locus frequency distributions. In this study, we constructed QTL–allele matrices for three plant-height-related traits (PH, NMS, and AIL) by including both positive and negative alleles from significant SNPLDB loci. The analyses showed that accessions with superior phenotypes carried positive haplotypes/alleles at higher frequencies than accessions with inferior phenotypes, and this pattern was evident in the QTL–allele matrices. The difference became most pronounced when we compared accessions with the highest and lowest phenotypic values. Notably, the organization of the QTL–allele matrix did not map directly onto results from traditional GWAS models.

The traditional single-locus GWAS analysis requires consideration of minor allele frequencies (MAFs). It is commonly believed that MAFs improve GWAS detection. However, in human genetics, significant loci identified by GWASs due to MAFs are often seen as pseudo-associations, resulting in false positives or negatives. To prevent such outcomes, loci with certain MAFs, typically 5% or 1%, were excluded in the single-locus GWAS analysis. This exclusion weakened the detection power of minor-effect genes, contributing to the phenomenon of missing heritability. Korte et al. (2013) suggested enhancing GWAS detection power by increasing sample size or refining the analysis algorithm [46]. In this study, we employed RTM-GWAS, a novel multi-locus GWAS method, to identify genetic loci associated with soybean plant height under varying light conditions. This approach replaced rare haplotypes (frequency of less than 0.01) with the most similar ones, thereby enhancing the detection power of common variants in the association analysis [22]. This method facilitated the discovery of genetic loci controlling traits, explaining why our previous single-locus GWAS detected fewer loci compared to RTM-GWAS. These findings aligned with the conclusions [44,47]. We used 181 soybean samples in this study, which increased the number of MAFs and potentially reduced detection power. However, RTM-GWAS improved the discovery of genetic loci related to plant height traits by altering the MAF distribution frequency.

Plant-height-related traits play a crucial role in soybean yield breeding and are governed by quantitative genes (loci). These quantitative traits are significantly influenced by environmental factors, complicating the detection of QTLs across diverse environments. Consistent identification of stable QTLs in various planting conditions is rare. In this study, QTLs detected in multiple environments were considered stable. Significant SNPLDBs identified in both multiple and single environments were considered stable QTLs for specific traits. Through a comprehensive analysis, four stable SNPLDB loci associated with plant height or internode length were identified using RTM-GWAS procedures. Of the 103 QTLs identified associated with plant-height-related traits under two light treatments across four environments, 99 were newly identified, and 4 were consistent with loci reported previously. A comparison with earlier studies showed that several significant loci overlap or lay near reported QTLs. For example, the locus LDB_2_48286454_48286476 on chromosome 2, which controls plant height under shade conditions, fell within the 45.83–48.29 Mb interval previously reported [48]. The shade-responsive locus LDB_13_6768908 mapped to 5.51–6.82 Mb, corresponding to a QTL described [49]. LDB_17_12390454_12391076 on chromosome 17 laid inside the physical intervals were reported [50,51]. Likewise, LDB_11_6477276_6522736 on chromosome 11, associated with NMS under normal light, was localized to 5.88–6.59 Mb. This interval coincided with the one reported by Chen et al. (2017) [52]. Thus, LDB_11_6477276_6522736 was likely the same QTL, via node number 3—3 described. Furthermore, the physical intervals of previously identified QTLs were significantly narrowed down in the current study, which indicated that the GWAS approach provided higher resolution than traditional linkage mapping. Linkage mapping relied on low-density markers and bi-parental mapping populations; therefore, previously reported QTLs had lower resolution [53]. Detecting QTLs with the high-resolution GWAS approach across multiple environments and models thus facilitates the development and implementation of marker-assisted breeding programs.

The primary objective of crop researchers is to identify the authentic functional genes located at genetic loci for immediate utilization in enhancing crop traits [54,55]. Despite the existence of functional genes related to plant height traits in soybeans, their availability has been undervalued, with only a limited number of genes characterized for height-related traits in soybeans [56,57]. In this investigation, through comprehensive gene annotation, a literature review, RNA-seq analysis, and an in silico examination, four candidate genes were identified within three stable genetic regions across various environments under different light conditions. These genes are considered potential candidates responsible for controlling plant height and average internode length in soybeans. They exhibit elevated gene expression in shoots during various developmental stages and are involved in numerous gene functions directly or indirectly linked to the regulation of plant height and development. These functions include those associated with intrinsic membrane components, cellular responses to xenobiotic and chemical stimuli, and negative regulation of gene expression. The candidate gene *Glyma13g38500* encodes beta-1,3-galactosyltransferase, which is involved in the modification of cell wall pectin, impacting intrinsic leaf size [58]. In addition, the gene *Glyma13g38630* encodes a WRKY transcription factor WRKY24 protein. Notably, mutations in its homolog (oswrky24) have been shown to significantly reduce plant height in rice [59]. Li et al. (2023) have reported an upregulation of *OsWRKY24* expression under conditions of darkness stress [60]. Furthermore, the gene *Glyma14g00530* encodes nitrate regulatory gene2, a crucial player in nitrate regulation [61,62]. It is well-documented that nitrate signaling pathways are associated with promoting plant growth, including influencing plant height [63,64]. Another candidate gene, *Glyma18g04580*, encodes a WYB transcription factor protein. Its homolog has been found to negatively regulate the inhibition of seed germination induced by abscisic acid (ABA) in Arabidopsis [65]. Previous research has highlighted the significant roles of plant hormones such as gibberellins (GAs), auxins, cytokinins (CKs), and ethylene in the regulation of plant-height-related traits [66,67,68]. Considering these findings, the four identified genes are promising candidates for influencing plant height or average internode length in soybean, particularly under varying light conditions. However, it is essential to conduct further functional validation tests to confirm the roles of these genes before considering their application in soybean breeding programs.

## 4. Materials and Methods

### 4.1. Plant Materials and Phenotypic Evaluation

A total of 181 soybean accessions from the soybean germplasm bank at Sichuan Agricultural University in China were selected to form a comprehensive association mapping panel. Names and origin of 181 soybean accessions are listed in Table S1. These accessions were cultivated in four distinct environments: E1 and E4 in Chongzhou (103°38′ E, 30°32′ N), Sichuan Province, during 2020 and 2022, and E2 and E3 in Renshou (104°09′ E, 30°00′ N), Sichuan Province, in 2021 and 2022. The study followed a randomized complete block design (RCBD) with three replications per environment. Two light conditions during the seedling stage were tested: normal light (PPFD = 1208 μmol/m^2^/s) and a shade condition (PPFD = 653 μmol/m^2^/s). The shade condition was induced using black shade nets that reduced light exposure by 50%, simulating the shading effect of intercropped maize on soybean seedlings. The red/far-red light ratio in spectral composition was 1.2~1.3 under normal light and shade conditions. After 35 days, the nets were removed to observe plant responses. Each accession was sown in three rows, with a row length of 1.5 m, a row spacing of 0.5 m, and a plant spacing of 0.1 m. Standard field management practices were uniformly applied across all plots.

The plant-height-related traits were measured at the maturity stage: plant height (PH), number of main stem nodes (NMS) and average internode length (AIL), under normal light and shade stress. The average value of the five plants was used as the trait observation. The determination of the index was carried out in accordance with the Specification and Data Standards for the Description of Soybean Germplasm Resources [69]. Plant height (PH) was defined as the total height from the main stem cotyledons to the apical growth point (cm). Number of main stem nodes (NMS) was defined as the count of internodes on the soybean main stem. Average internode length (AIL) was calculated as the average length of main stem internodes, determined by dividing plant height by number of nodes (cm). Descriptive statistics were calculated and an analysis of variance (ANOVA) was conducted for the three plant-height–related traits using IBM SPSS 26.0 software. Broad-sense heritability (*h*^2^) for the three traits across environments were estimated using QTL IciMapping 4.1.

### 4.2. Genotyping and SNPLDB Construction

Genomic DNA was extracted from fresh leaves at the four-leaf stage according to the cetyltrimethylammonium bromide (CTAB) method [70]. The Genomic DNA samples were sent to MolBreeding Biotechnology Ltd. (Shijiazhuang City, China) for genotyping using the Soybean40K liquid phase chip platform [71]. After filtering with the criteria of a minor allele frequency (MAF)  >  0.05 and a missing rate  <  10%, 77,159 markers were obtained.

To establish a marker system with multiple haplotypes/alleles, we utilized SNP linkage disequilibrium blocks (SNPLDB) determined through the confidence interval method. Haploview v 4.2 was employed with a default configuration, except for adjustments made to the maximum distance and minimum minor allele frequency (MAF) parameters, which were set to 200 kb and 0.01, respectively [72]. SNPLDBs can encompass multiple SNPs with various haplotypes/alleles or may consist of a single SNP with two haplotypes/alleles. In cases where the allele frequency of a haplotype is below 1%, it is substituted with an approximate haplotype with the highest frequency.

### 4.3. Marker–Trait Associations via RTM-GWAS

We applied the RTM-GWAS procedure, a two-stage association analysis, to investigate three plant-height-related traits (PH, AIL, and NMS) across four distinct environments under normal light and shade conditions, using implied SNPLDBs with multiple alleles [22]. SNPLDBs exhibiting a significance level of *p* < 0.05 were considered as significant loci in the two-stage association analysis of the RTM-GWAS procedure to identify genome-wide QTL alleles, consistent with previous studies (Meng et al., 2016; He et al., 2017) [21,22]. For the significant SNPLDB loci associated with the target traits, allelic effect values were computed in the second-stage association analysis of the RTM-GWAS procedure [22]. Consequently, the negative and positive alleles of all significant SNPLDB loci were determined based on the allelic effect values.

### 4.4. Haplotype Analysis and Frequency Distribution of the Major SNPLDB Loci

In this study, significant loci were pinpointed using a multiple-environment combined model and at least one single-environment model, showing high values (−lg(*p*) ≥ 10.00) and explained phenotypic variance (PVE ≥ 1.00%). These loci were categorized as major stable SNPLDB loci. The average phenotypic value for each haplotype/allele at these major stable SNPLDB loci was calculated. A two-tailed T-test was performed using SPSS 26.0 software to assess the significance between the two alleles, identifying the favorable types of haplotype/allele variation. Box plots depicting the phenotypic values associated with the alleles were created using R 4.2.1. From the 181 soybean accessions, the 30 accessions with the highest phenotypic values (tall accessions) and the 30 accessions with the lowest phenotypic values (dwarf accessions) were chosen for further examination. The frequencies of PH/AIL were monitored under normal light and shade conditions.

### 4.5. Mining Potential Candidate Genes Controlling Plant-Height-Related Traits

The physical locations of SNPLDB loci that exhibited significant associations with the target traits were compared meticulously with all genes cataloged in the Soybase database (https://soybase.org). To explore further, the genomic regions surrounding the SNPLDB loci were scrutinized within a 180 kb span of the soybean reference genome (Wm82G1yma v1.0), examining both upstream and downstream decay distances. Heatmaps that illustrated gene expression profiles across diverse tissues were constructed by amalgamating RNA-seq expression data sourced from the SoyMD database (https://yanglab.hzau.edu.cn/SoyMD (accessed on 21 May 2024)), which facilitated the initial screening process. Furthermore, to enhance the precision of gene selection, the amino acid sequences were aligned with the Arabidopsis thaliana database to identify candidate genes that might contribute to plant height development, leveraging homologous gene functions for this identification.

### 4.6. Candidate Gene Validation

Based on the plant height phenotypes under two light treatments, ZY121 with a lower PH and ZY153 with a higher PH were selected from the 181 accessions. qRT-PCR was carried out to examine the relative expression of candidate genes in these two cultivars. Plants were grown in the Conzhou experimental environment, and stem samples were collected at 15, 25, and 35 days after germination. Total RNA was extracted using the Trizol method (Omega, Bio-tek, Shanghai, China), and cDNA was synthesized with a PrimeSTAR^®^ GXL DNA Polymerase kit (Takara. Bio Inc., Kusatsu, Shiga, Japan). qRT-PCR analysis was performed on a CFX Opus real-time fluorescence quantitative PCR system (BIO-RAD, Hercules, CA, USA), with Gmtubulin4(XM_003554060) serving as the housekeep gene for normalization. Primers (Table S8) were designed with Primer5.0, and reactions used the UltraStart Universal SYBR qPCR Master Mix kit (Exongen, Chengdu, China). Each 10 μL reaction contained 1 μL diluted cDNA, 0.8 μL forward and reverse primers, 3.2 μL ddH_2_O, and 5 μL 2 × Ultra SYBR Mixture. The whole reaction was run under the following conditions: pre-denaturation at 95 °C for 3 min; PCR for 40 cycles, 95 °C for 30 s, 60 °C for 30 s, 72 °C for 20 s; solubility curve analysis at 95 °C for 20 s, 65 °C for 30 s, and 96 °C for 1 s. Relative gene expression was calculated by the 2^−ΔΔCT^ method, and the primer sequences for both the candidate gene and the reference gene are listed in Table S3. For each gene, we performed three biological replicates and three technical replicates.

## 5. Conclusions

This study revealed a total of 42 significant SNPLDB loci associated with plant height (PH), 33 with average internode length (AIL), and 28 with number of main stem nodes (NMS) within a panel of 181 soybean accessions by the RTM-GWAS procedure conducted under both normal light and shade conditions over a three-year period. The effects of haplotypes and alleles at these loci were meticulously calculated, leading to the creation of QTL–allele matrices. These matrices provided a concise representation of the genetic composition of the soybean panel. Among the loci identified, four were consistently detected across multiple environments and were classified as stable SNPLDB loci under normal light and shade conditions. These four stable loci were selected for further investigation. A thorough analysis identified 80 genes annotated near these loci. Using an integrated approach that included gene annotations, an in silico analysis, an RNA-seq analysis, a literature review, and qRT-PCR, four potential candidate genes likely regulating the target traits associated with these stable loci were detected. This study not only revealed QTL alleles linked to plant-height-related traits in soybean but also highlighted potential candidate genes that play crucial roles in these traits. The findings offer valuable insights into the genetic basis of plant-height-related characteristics in soybean under shade stress, significantly contributing to the understanding of soybean genetics and breeding strategies.

## Figures and Tables

**Table 1:** Statistic descriptions of three height traits for four different environments in 181 soybean accessions.

Traits	E	T	Min	Max	Mean	SD	CV (%)
PH (cm)	E1	N	14.82	44.62	25.74	6.41	24.9
		S	26.07	102.2	58.58	17.76	30.32
	E2	N	29.08	73.08	45.38	7.68	16.92
		S	39	91.5	59.46	10.39	17.47
	E3	N	34.92	108.83	57.58	12.98	22.54
		S	45.92	124	79.23	15.47	19.53
	E4	N	22.5	70.5	37.9	9.36	24.7
		S	34.58	113.67	63.92	15.35	24.01
NMS	E1	N	5	8.5	6.59	0.7	10.62
		S	3.83	8.67	6.27	0.83	13.24
	E2	N	7.17	12.17	9.8	0.89	9.08
		S	7.83	12.33	10.25	0.83	8.1
	E3	N	9	15.67	12.23	1.33	10.87
		S	9	16.17	12.97	1.42	10.95
	E4	N	6.17	12.83	9.25	1.35	14.59
		S	7.67	13	10.64	1.07	10.06
AIL (cm)	E1	N	2.22	6.77	3.92	0.84	21.43
		S	4.23	16.25	9.5	2.46	25.89
	E2	N	3.2	6.53	4.63	0.63	13.61
		S	3.72	8.6	5.84	0.93	15.92
	E3	N	3.08	7.25	4.69	0.83	17.7
		S	4.02	9.34	6.1	1	16.39
	E4	N	2.53	6.38	4.1	0.77	18.78
		S	3.75	9.71	5.99	1.15	19.2

Note: E represents environment; T represents treatment; Max, maximum; Min, minimum; SD, standard deviation; CV, coefficient variation. N: normal light; S: shade condition.

**Table 2:** Variance analysis for three height-related traits assessed in 181 soybean accessions.

Traits	Source	df	SS	MS	F	*p*-Value	*h*^2^ (%)	
							N	S
PH	E	3	246,838.82	82,279.61	8491.66	0.000	65.73	60.38
	T	1	398,412.58	398,412.58	41,118.14	0.000		
	G	180	277,165.54	1539.81	158.92	0.000		
	E × T	3	33,682.04	11,227.35	1158.72	0.000		
	E × G	540	75,808.07	140.39	14.49	0.000		
	T × G	180	29,266.64	162.59	16.78	0.000		
	E × T × G	527	54,398.40	103.22	10.65	0.000		
NMS	E	3	13,672.47	4557.49	8840.04	0.000	34.77	29.81
	T	1	220.53	220.53	427.75	0.000		
	G	180	1318.30	7.32	14.21	0.000		
	E × T	3	270.35	90.12	174.80	0.000		
	E × G	540	850.82	1.58	3.06	0.000		
	T × G	180	317.18	1.76	3.42	0.000		
	E × T × G	527	858.44	1.63	3.16	0.000		
AIL	E	3	1160.63	386.88	2097.24	0.000	63.84	48.82
	T	1	4449.12	4449.12	24,118.54	0.000		
	G	180	2203.76	12.24	66.37	0.000		
	E × T	3	2130.36	710.12	3849.54	0.000		
	E × G	540	819.86	1.52	8.23	0.000		
	T × G	180	311.60	1.73	9.38	0.000		
	E × T × G	527	605.80	1.15	6.23	0.000		

Note: E represents environment; T represents treatment; G represents genotype; E × T, E × G and T × G respectively represent the interaction between environment and treatment, the interaction between environment and genotype, and the interaction between treatment and genotype. E × T × G represents the interaction between environment, treatment and genotype. *h*^2^, broad-sense heritability.

**Table 3:** The summary of the number of SNPs and LDBs located on 20 chromosomes of soybean.

Chr.	SNP	LDB	Chr.	SNP	LDB
1	3821	559	11	2900	461
2	3887	558	12	1814	249
3	4071	702	13	4162	655
4	3434	562	14	3918	550
5	2786	408	15	5052	611
6	4481	664	16	3624	660
7	4039	600	17	4288	543
8	4209	470	18	7093	1040
9	3648	520	19	3564	539
10	3274	539	20	3446	573
Total	37,650	5582		39,861	5881

**Table 4:** The significant LDBs associated with three soybean height traits in multiple and at least one single test environments.

Traits	T	LDB Loci	Chr	Position	−lg10 (*p*)	PVE (%)	Single Test Environments
PH	N	LDB_14_228886	14	228,886	10.27	1.76	E3 (2.78)
		LDB_13_39157370_39256624	13	39,157,370	24.03	1.7	E1 (2.27), E2 (8.31), E4 (3.87)
		LDB_4_44306173_44337361	4	44,306,173	4.44	0.95	E1 (7.81)
		LDB_3_32845250_32871460	3	32,845,250	6.11	0.50	E3 (2.06)
	S	LDB_13_39157370_39256624	13	39,157,370	18.23	2.14	E2 (6.03), E3 (5.18), E4 (11.49)
		LDB_8_21969552	8	21,969,552	3.66	0.66	E4 (3.23)
		LDB_2_4137092	2	4,137,092	5.31	0.39	E3 (5.69)
NMS	S	LDB_9_36844563	9	36,844,563	8.16	0.38	E3 (10.7)
		LDB_18_61052742_61052771	18	61,052,742	4.52	0.31	E3 (6.61)
AIL	N	LDB_3_28962185_29060976	3	28,962,185	7.66	2.06	E1 (19.30)
		LDB_18_3424413_3513390	18	3,424,413	13.01	1.53	E4 (15.74)
		LDB_16_6449440_6483302	16	6,449,440	7.45	1.03	E3 (12.99)

## Data Availability

The relevant data of the paper are provided in the Supplementary Materials.

## Supplementary Materials

The following supporting information can be downloaded at: https://www.mdpi.com/article/10.3390/ijms27125598/s1.
